#  Analysis of Genome-scale Expression Network in Four Major Bacterial Residents of Cystic Fibrosis Lung

**DOI:** 10.2174/1389202915666140818205444

**Published:** 2014-10

**Authors:** Nazanin Hosseinkhan, Peyman Zarrineh, Ali Masoudi-Nejad

**Affiliations:** Laboratory of Systems Biology and Bioinformatics (LBB), Institute of Biochemistry and Biophysics, University of Tehran, Tehran, Iran

**Keywords:** Lung resident organisms, Differentially expressed genes, Cystic fibrosis.

## Abstract

In polymicrobial communities where several species co-exist in a certain niche and consequently the possibility of interactions among species is very high, gene expression data sources can give better insights in to underlying adaptation mechanisms assumed by bacteria. Furthermore, several possible synergistic or antagonistic interactions among species can be investigated through gene expression comparisons. Lung is one of the habitats harboring several distinct pathogens during severe pulmonary disorders such as chronic obstructive pulmonary disease (COPD) and cystic fibrosis (CF). Expression data analysis of these lung residents can help to gain a better understanding on how these species interact with each other within the host cells. The first part of this paper deals with introducing available data sources for the major bacteria responsible for causing lung diseases and their genomic relations. In the second part, the main focus is on the studies concerning gene expression analyses of these species.

## RESIDENT MICROORGANISMS IN LUNG

Microbial communities (also known as microbiome) belong to distinct parts of human body; consist of complex collection of microorganisms which usually exist in polymicrobial communities. The large number of microorganisms occurring in a small space leads to a huge number of possible interactions among the cohabitants in that community which leads to a so called “social evolution”. The range of the relationships within a community can be very diverse from mutualistic to commensalistic to antagonistic types. In addition, some interactions involve direct physical contacts while others comprise cell-cell signaling through quorum-sensing cross-talk [1]. A particular example is the human respiratory tract, which as a consequence of its continuous exposure to the external environments, can be occupied by a variety of pathogenic microorganisms. This in turn leads to a variety of human-microbe and microbe-microbe interactions [2]. This is obviously observed in severe respiratory tract disorders such as chronic obstructive pulmonary disease (COPD) and cystic fibrosis (CF), in which mucocilliary clearance and lung innate immunity have been impaired. The resulted situations simultaneously lead to the presence of a wide range of microorganisms in the lung which is responsible for the high levels of morbidity and mortality observed in these diseases. Based on the traditional culture based microbiological

diagnostic tests, the commonly reported microorganisms in lung diseases include *Pseudomonas aeruginosa*, *Burkhol- deria cepacia* complex (most frequently *B.cenocepacia* spps), *Staphylococcus aureus*, *Haemophilus influenzae*, *Alcaligenes xylosoxidans*, *Stenotrophomonas maltophilia *and *Candida albi*cans. However, thanks to the advancement of the molecular techniques, more diverse range of microorganisms have been recorded recently comprising species of *Veillonella*, *Streptococcus*, *Abiotrophia,* *Gemella*, *Neisseria* and *Acinetobacter*. As previously mentioned this microbial diversity causes many interactions that have influences on the pathology of the members of a given community and their response to the antibiotic treatments [3]. It has been postulated that microbial communities such as those observed in CF lung act as a pathogenic entity rather than the combination of individual members [4].

During the last century, investigating microorganisms’ behavior under the certain laboratory conditions was the main focus of most microbiological studies. This has provided a wealth of information about the physiological and genetic basis of organisms that in turn has facilitated rapid advances in molecular biology. However it is now widely accepted that reasons for ecological success of bacteria in complex environments such as polymicrobial communities can precisely be investigated through considering all possible interactions existing among them [5].

The genetic basis of the reactions mediated by microorganisms was traditionally studied through investigating the expression of single genes or cluster of genes using different experimental methods. However owing to the development of high throughput gene expression analysis techniques, inspection of global gene expression under a variety of conditions has recently become possible [6]. In this article we have tried to introduce available data sets for the most important CF lung bacteria, comparing the genome content of these bacteria, highlighting the gene expression data source values in the studies addressing CF lung infections, and reviewing the papers concerning with differentially expressed gene sets under CF lung conditions.

## BACKGROUND ON UNDERLYING BIOLOGICAL PROCESSES OF BACTERIAL SPECIES FOUND IN LUNG INFECTIONS

Occurrence of infectious diseases involves regulation of genes expressions in response to the host environment. Bacterial infections are also the direct result of interactions among pathogens residing in the host. Inside human body, bacterial pathogens not only interact with host and other pathogens, but also communicate with the normal, non-pathogenic microflora [7]. This is especially true in the case of lung infections such as CF disease. This happens in spite of the hostile nature of human respiratory airways for bacteria. Nutrients and oxygen limitation and the presence of several antibacterial agents in human airways during lung infections, make it a threatening environment for most of the pathogenic bacteria. In the case of chronic respiratory tract infections as described above, as a consequence of co-existence of several pathogenic microorganisms (most notorious ones as mentioned earlier are *P. aeruginosa*, *S. aureus*, *H. influenza* and *B. cenocepacia*) beside normal residents of lung, the proper function of lung is impaired. This subsequently leads to the high mortality and morbidity associated with this disease (General information about the main pathogens of lung has been represented in Table **1**). The pathogenesis of polymicrobial communities such as those observed in CF is associated with molecular interactions and cell-cell communication through quorum sensing. Quorum sensing is a density dependent cell–cell signaling system which can coordinate gene expression in a bacterial community using small diffusible molecules called autoinducers. This mechanism helps bacteria to communicate with each other and coordinate their behavior in bacterial societies [8]. *N*-Acyl homoserine lactones (AHLs or N-AHLs) that constitute a class of signaling molecules are the major autoinducers, participating in quorum sensing. Role of AHLs in virulence factors production in human pathogens has been indicated [9]. It has also been shown that some autoinducers act as interspecies signaling molecule [10, 11]. This can be an explanation for observed synergistic relationships among bacteria. This phenomenon which is referred to as “cross-talk” between bacteria, first recorded in gram negative bacteria. For example AI-2 autoinducer of LuxS quorum sensing system belonging to *P. aeruginosa *has been shown to play a major role in mixed-species biofilm formation [12] that is considered as an important contributor to persistent respiratory tract diseases [13]. It has also previously been indicated that *P. aeruginosa* and *B. cenocepacia* are able to utilize heterologous quorum sensing signaling molecule which has been proposed to play roles in coordinately regulation of some virulence factors in these bacteria. In polymicrobial communities like those observed in biofilms, bacterial species experience changes in selection pressure that occurs as a consequence of different factors including host immune response, antibiotic pressure, low availability of nutrients and oxygen limitation [14]. It has frequently been shown that biofilms are usually composed of multiple distinct species. The observed heterogeneity in species found in biofilms has a great contribution in both recurrent and persistent infections [15]. Examples of *P. aeruginosa*-*S. aureus* [16], *P. aeruginosa*-*B. cenocepacia* [17] and *P. aeruginosa*-*H. influenzae* mixed biofilms, have been previously described.

## LUNG CO-INFECTIONS

**Fig. (1):**
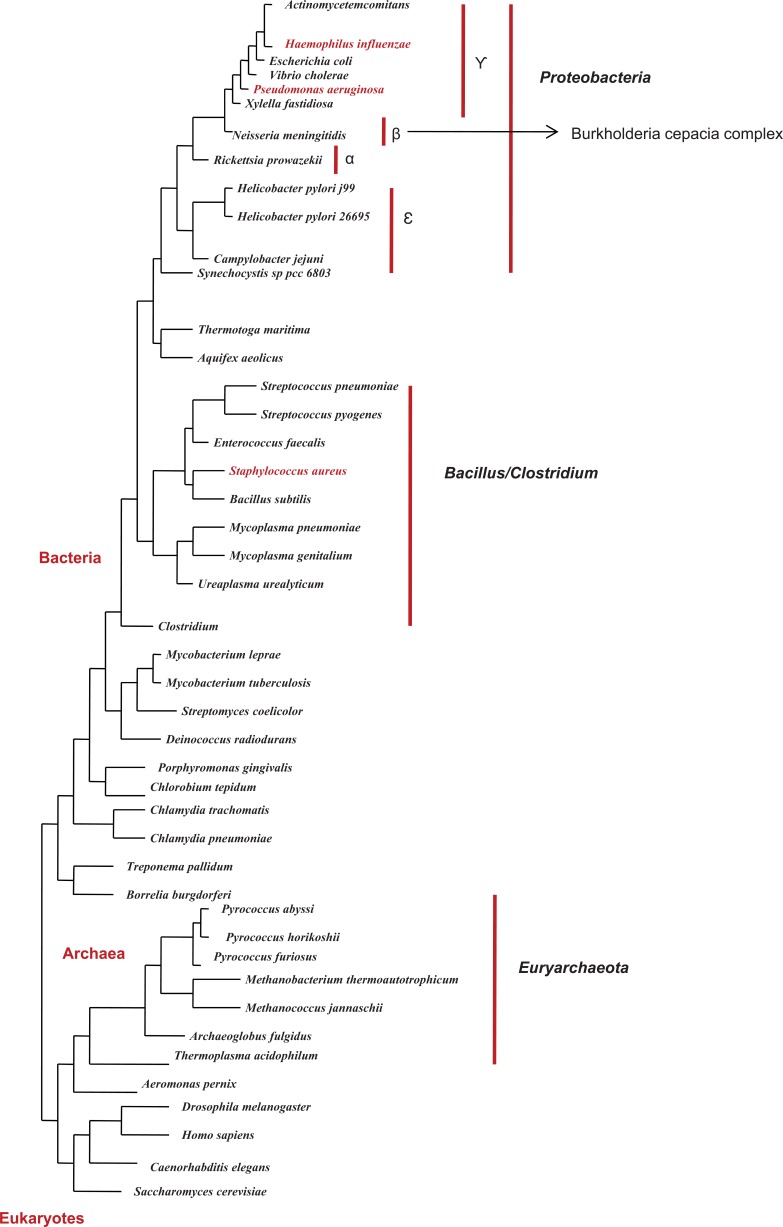
Distribution of *P. aeruginosa, H. influenza, B. cenocepacia* and *S. aureus* across bacterial phylogenetic tree. *P. aeruginosa, H. influenza *and* B. cenocepacia* belong to group of proteobacteria (placed in the vicinity of each other in tree) while *S. aureus* is distantly related to them and belong to bacillus and clostridium group.

As mentioned above, several bacterial species can simultaneously reside in the lung during severe lung infections. Several cases of co-infections have been so far reported in CF lung infections, in which *P. aeruginosa, S. aureus, B. cenocepacia *and* H. influenzae* co-infections are the most prominent ones. Phylogenetic distribution of mentioned pathogens across bacterial phylogenetic tree has been represented in (Fig. **1**). Studying interactions between these organisms in the lung at the molecular level can elucidate fundamental characteristics associated with concomitant infections.

**Fig. (2):**
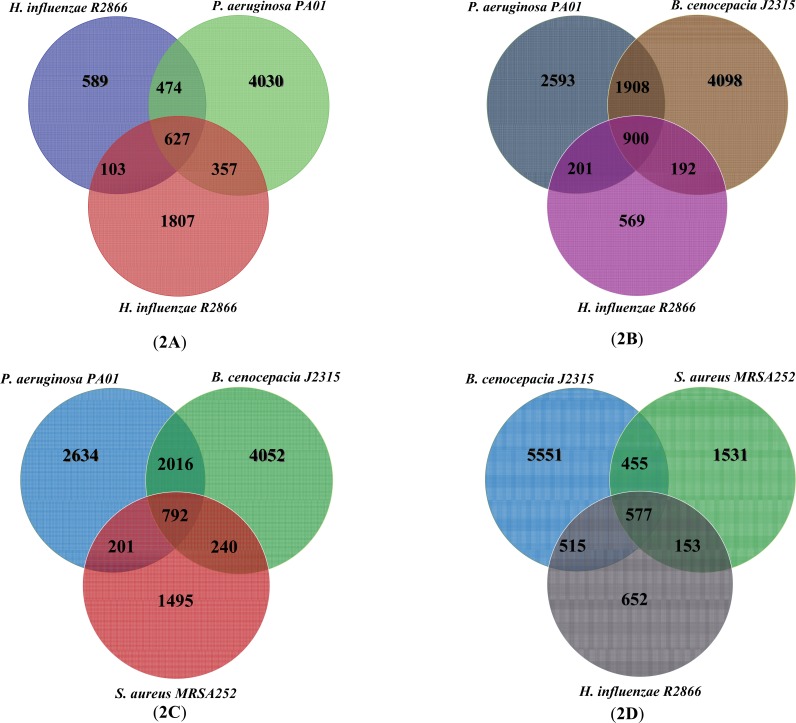
Comparative orthologous genes analysis of four dominant lung pathogens: *P. aeruginosa* *PAO1, S*. *aureus MRSA252*, *H. influenzae R2866 *and *B. Cenocepacia J2315*. Venn diagrams show shared and species specific orthologous genes in four species. The observed discrepancy between the number of genes for each bacterium presented in Table **1** with the sum of the number of genes in Venn diagrams shown in Figure **2** is due to this fact that the number of genes in the Table **1** are the sum of the genes present in all strains of that bacterial species whereas in Figure **2** the data is for only one strain.

Numbers of orthologous genes which are common between *P. aeruginosa, S. aureus, B. cenocepacia *and* H. influenzae* have been shown in (Fig. **2**). By definition orthologous genes are homologous genes (genes with significant sequence conservation) diverged from a common ancestor through speciation events. Available gene expression, protein interaction and metabolic network data of the four species have been represented in (Table **2**).

## USING EXPRESSION DATA TO DETECT DIFFERENTIALLY EXPRESSED GENES AND CO-EXPRESSION CONSERVATION

Most available data sources such as protein-protein and regulatory interaction networks are static interaction 

measurements which cannot highlight the changes in the cell life under different laboratory conditions. Dynamic responses of a biological system to the environmental changes can be portrayed either through gene expression data sources or metabolic fluxes [25].

Gene expression studies have contributed largely in gaining deeper knowledge about the regulation and function of genes in various domains of biological studies including developmental, physiological and pathological processes [26]. Since the pattern of gene expression of many genes may be changed under the conditions experienced by the cells, several distinct studies have been conducted to investigate the expression behavior of cells or tissues under a variety of conditions. In the case of pathogenic bacteria, conducting differential expression analysis can contribute to the detection of over-expressed or under-expressed genes under pathogenicity related conditions.

**Fig. (3):**
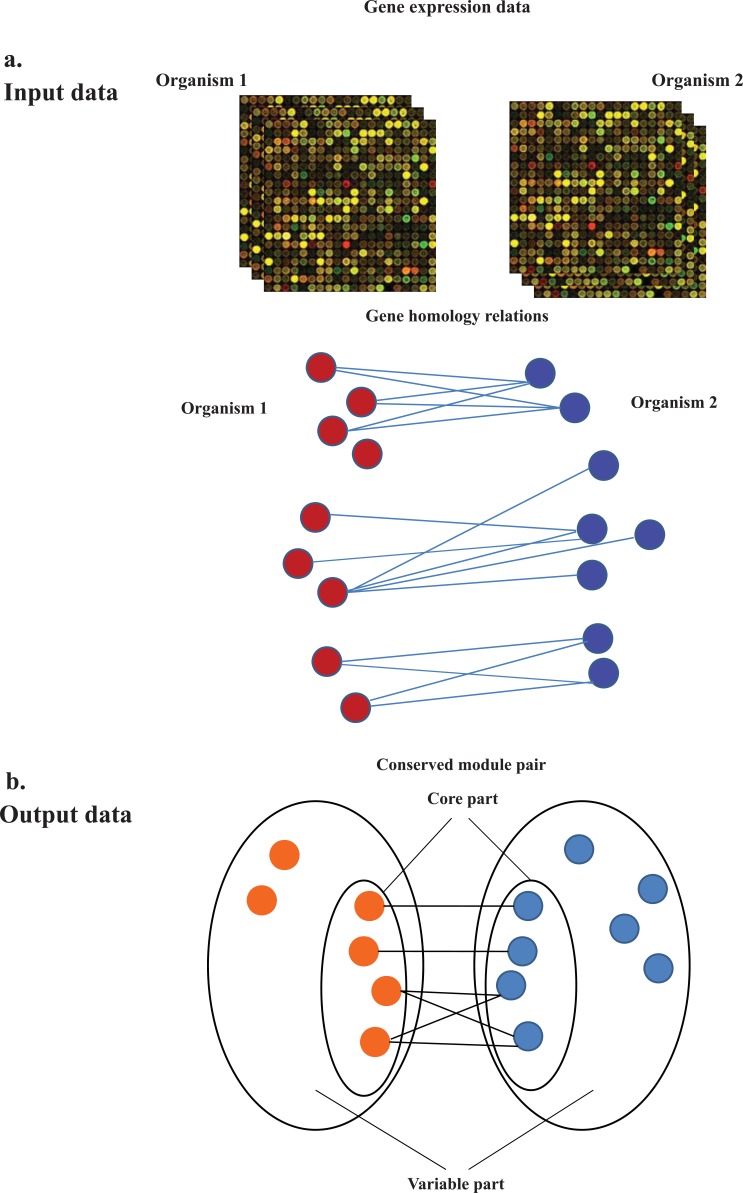
Comparative gene co-expression **A.** Input data includes gene expression data sources for understudied species as well as the list of homologous genes identified by sequence similarities. **B.** The output data usually consist of core part corresponding to the common homologous genes that link up both co-expression modules and for which the mutual co-expression behavior is conserved between two species and the variable part which is composed of species-specific genes [27].

The conservation of expression profiles between orthologous genes is often compared in cross-species gene expression analysis. The conservation of expression will increase the chance of exhibiting similar function by orthologous and/or homologous genes across species. In most recent studies the scope of conservation has been expanded to homologous expression conservation. This is partly due to this fact that in many cases homologous genes may assume similar functions across species [27]. Furthermore, in bacterial evolution studies the evolutionary description of orthology is somewhat different and this is because of the high rate of horizontal gene transferring (HGT) that is a commonly observed phenomenon in bacteria. In HGT foreign genes are acquired by organisms and it has been shown that many homologous genes in bacteria have been resulted from HGT rather than from orthology relationship. Hence these genes are referred to xenologous genes. However finding all instances of HGT and confirming it is not straightforward. Consequently, inferring functional correspondence is a challenging task as it is commonly deduced from observed sequence similarity by methods like BLAST [28]. Comparative gene co-expression studies (Fig. **3**) are among the most widely used methods for inspecting expressing genes. These types of studies are conducted with the aims of specifying genes associated with certain conditions including different types of tissues, organisms or cancers. Some available functional data have been inferred based on sequence similarity between genes that is not much reliable. As emphasized earlier, genes showing co-expression in different conditions as a result of their transcriptional coordination, tend to be involved in the similar biological process [29]. This subsequently will help to infer the probable functions of genes already having uncharacterized functions. As a comparative co-expression study example in bacteria, two mostly studied bacteria; *Escherichia coli* and *Bacillus subtilis* co-expressed modules were compared [27]. In this study, each detected conserved co-expressed module consisted of a core part and a variable part (see Fig. **3**). The core part comprised of the homologous genes and the variable part contained the additional genes in each co-expressed modules. It has been shown that the variable part of the conserved co-expressed modules largely included species specific genes, indicating that bacteria are also flexible in adding new members to an existing co-expressed module. Consequently performing comparative studies among distinct organisms, which can help to characterize species specific functions vs. conserved ones, can be a more powerful tool for completing functional data.

Northern blotting, Reverse Transcription quantitative PCR (RT-qPCR), Real time quantitative PCR, Sequential Analysis of Gene Expression (SAGE), DNA microarray , RNA-Seq and expressed sequence tags(ESTs) are among the most widely used experimental techniques frequently applied to measure genes expression levels. The advantages and disadvantages of three highly used techniques (Microarray, SAGE and RNA-Seq) have been compared in (Table **3**). It is worth mentioning here that microarray technique has been the most broadly used methods in gene expression analysis studies until recently, but the trend is shifting from microarray to RNA-Seq technique.

## PSEUDOMONAS AERUGINOSA

*P. aeruginosa* is a gram negative bacilli belonging to gamma group of proteobacteria. Its genome is one of the largest among other bacterial genomes (Table **3**).* P. aeruginosa* has more regulatory genes than other bacteria. These include genes involved in two-component signal transduction systems, transcriptional regulators and transport systems. *P. aeruginosa *is able to survive in a variety of ecological conditions due to containing a large number of transcriptional regulators and two-component regulatory systems. *P. aeruginosa *possesses about 150 outer membrane proteins that have been suggested to play significant roles in antibiotic resistance, iron uptake and secretion of virulence factors. This microorganism is also able to degrade a variety of substances comprising carbonic acids, sugars, fatty acids, alcohols, polyalcohols, glycols, aromatic compounds, amines and amino acids [33]. All mentioned characteristics beside its ability to produce biofilm make *P. aeruginosa* a successful opportunistic pathogen with the ability to survive and growth in various ecological conditions. In addition to being a causative agent of pneumonia, urinary tract and wound infections, *P. aeruginosa* can produce infections in immune-compromised patients such as AIDS and cancer patients. Moreover *P. aeruginosa* has a clear role in the morbidity and mortality associated with CF which as previously described, is an autosomal recessive genetic disorders caused by mutation in a chloride channel known as Cystic Fibrosis transmembrane Regulator (CFTR). As a result of production of dehydrated viscous mucous in the lung of cystic fibrosis patients, the airways is very susceptible to become simultaneously colonized with other pathogens beside *P. aeruginosa*. *P. aeruginosa* can out-compete other species present in cystic fibrosis lung including *B. cenocepacia*, *S. aureus* and *H. influenzae*. However not much is known about the underlying mechanisms making *P. aeruginosa *able to do this.

A few studies have addressed the expressional responses of *P. aeruginosa*. In a study conducted by Duan and co-workers the effect of oropharyngeal flora (OF), comprising *Streptococcus* and *Staphylococcus* strains, on *P. aeruginosa* virulence was investigated through performing genome wide gene expression profiling of *P. aeruginosa* in the presence of OF strains. Results showed that OF have a great influence on *P. aeruginosa* via modulation of its gene expression. Results demonstrated that in total 214 and 171 *P. aeruginosa* promoters were respectively affected by *Streptococcus* and *Staphylococcus *strains. Their results also showed that *P. aeruginosa* genes modulated by OF belong to a variety of groups including virulence, transporter or membrane, metabolism, iron uptake or storage, transcriptional regulators and secreted proteins [7]. In another study carried out by the same research group, the expression profiles of 31 promoter-reporter set in *P. aeruginosa *strains under a vast variety of conditions (72 conditions) was measured. Subsequently according to the co-expressed (co-regulated) genes, the 

conditions were classified into a number of groups. The interaction network was then constructed in any individual conditions [34]. In the recent study Thøgersen and colleagues tried to cluster five gene expression datasets of *P. aeruginosa* strains isolated from the lung of cystic fibrosis patients under diverse conditions including growth medium and growth state. Bacterial isolates belonged to different clinically relevant phenotypes like mucoidy, and hypermutability. In this study Archetypal Analysis (AA) method which is a clustering technique benefited from two popular expression clustering methods known as K-means clustering and principle component analysis (PCA), was employed. AA is based on finding a few representative points in the datasets. Combination of these points (archetypes) can describe the entire dataset. This in turn will help in dimension reduction of large datasets. Thøgersen and colleagues could identify seven archetypes which were able to extract the main biological features, *i.e.* genes experiencing changes in their expression during adaptation phase to the cystic fibrosis lung environment in the studied datasets [35]. Archetypes were first specified based on their specific gene profiles (genes representing remarkable changes in their expressions). After identification of archetypes it is possible to see how samples are clustered based on archetypes. The identified genes were then assigned to 26 gene ontology (GO) class previously defined for *P. aeruginosa*. In the next step overrepresented GO classes in the up-regulated or down-regulated genes were specified using hypergeometric distribution test. Finally using AA method, different datasets were clustered in to biologically meaningful groups. For instance archetypes 1, 2 and 5 could separate one of the datasets (study2) in to two groups representing early and late (isolated from early and late stage of infection respectively) isolates. Early strains characterized by Archetype1 showed high expression of genes belonging to the GO class “Motility and Attachment” and a low expression of genes related to “Amino acid biosynthesis and metabolism”. On the other hand late strains portrayed by archetype 2 are characterized by up-regulation of genes related to “Antibiotic resistance and susceptibility”, “Two-component regulatory systems” and genes “Related to phage, transposon and plasmid” while down-regulated genes belong to the functional classes “Adaptation, Protection” and “Secreted factors”. Genes represented by archetype 5 were involved in alginate biosynthesis and belong to the GO class of “secreted factors”.

## BURKHOLDERIA CEPACIA COMPLEX

*Burkholderia cepacia* complex (Bcc) comprises at least 17 taxonomically related gram negative species (Strains of *B. cenocepacia* have been frequently isolated from Lung infections specially from CF). The size of Bcc genomes are one of the largest among other bacterial genomes and are comparable with *P. aeruginosa* (Table **1**). They are frequently found in natural environments, and they are also considered as opportunistic pathogens in immunocompromised patients and in CF lung. Members of Bcc often co-exist with *P. aeruginosa* in soil and also in the lung of CF patients [36]. *B. cenocepacia *is a member of Bcc which frequently isolated from patients with CF, accounting for 50% to 80% cases of infection [37]. Colonization of CF lung by Bcc is threatening and will cause in progressive worsening of lung function and consequently the increased risk of death in these patients. This high risk is to some extent associated with high degree of reported antibiotic resistance in Bcc. Worgall and colleagues tried to understand the interaction of human Alveolar Macrophages (AM) obtained from smoker and non-smoker individuals with *P. aeruginosa *and *B. cenocepacia* strains through evaluation of the early changes in AM gene expression [38]. Their result indicated that while AM has a vital role in inflammatory response of human respiratory epithelium; it shows similar response to different* P. aeruginosa *and *B. cenocepacia* strains. In another study conducted by P. Mira *et al*. genome wide gene expression analysis was performed to understand different adaptive strategies assumed by *B. cenocepacia* during chronic colonization in CF lung, [14]. In this study transcriptome of two *B. cenocepacia* strains obtained from CF lung in initial and chronic stages (3 years after initial colonization) of infection were compared. Using 1.5-fold threshold value, 1024 genes were differentially expressed in these two variants, 534 and 490 up-regulated and down-regulated respectively. The same trend has also been demonstrated for *P. aeruginosa.* It has been indicated that Bcc infected CF patients show more reduced life span than non-Bcc infected ones. It may partly be due to the high degree of antibiotic resistance in these strains. To characterize all genomic features concerned with Bcc virulence, R. Yoder-Himes and colleagues made a comparison between the transcriptome of an epidemic strain of *B. cenocepacia*, under the same condition as CF sputum, and the transcriptome of an environmental (soil) strain. They showed that genes involved in intracellular trafficking and translation represent higher expression under conditions mimicking CF lung; whereas genes responsible for signal transduction or amino acids and carbohydrates metabolism show up-regulation in environmental conditions [39].

## STAPHYLOCOCCUS AUREUS

*S. aureus* is a gram positive coccus that is often found as a commensal on skin surface, skin glands and mucus membranes. 20 to 30% of human population has been estimated to be carriers for *S. aureus *[40]. However when *S. aureus* passes over the epithelium and gain access to internal parts of the body turns to a threatening pathogen that produces a collection of different virulence factors. The production of biofilm is also reported in some case of *S. aureus* infections [41]. *S. aureus* can adapt itself to antibiotic therapy during long term infections. Mechanisms involved in antibiotic resistance include up-regulation of bacterial efflux pumps and mutation of antibiotics target molecules. Moreover transforming to small colony variant (SCV) forms or growth in biofilms play significant roles in the observed antibiotic resistance [42]. Some expressional analysis performed on *S. aureus *will be discussed in brief here. 

Weinrick *et al*. examined the expressional responses of *S. aureus *in mild acidic pH resulting from glucose fermentation. They described down-regulation of some virulence genes in *S. aureus* in pH 5.5 [43]. In another study, Resch and colleagues conducted comparative expression analysis on *S. aureus *cultivated under biofilm and planktonic conditions. Their results showed up-regulation of secreted, cell wall, transporter, ribosomal and physiological proteins in biofilm conditions [44]. Cui and colleagues also made a comparison between the expression profiles of vancomycin intermediate (VISA) and vancomycin susceptible (VSSA) strains of *S. aureus *to find genes involved in glycopeptide resistance. They found seventeen genes responsible for increasing the resistance of *S. aureus* to glycopeptides through their over expression. Fourteen out of seventeen genes which reduced the susceptibility to glycopeptides belong to two broad functional categories: 1- envelope and cellular processes and 2- regulators [45]. In a study carried out by Moisan and colleagues, gene expression profiling of *S. aureus* small colony variants (SCVs) produced during persistent infections, were compared with wild-type prototype strain and hemin biosynthetic gene mutant (hemB). They could determine differentially expressed genes responsible for conversion of wild type strains to SCVs; 20 and 22 genes were respectively up-regulated and down-regulated [46]. In another study Rogasch *et al*. assessed the contribution of two component system SaeRS on global transcriptional responses of *S. aureus*. They showed that while the expression of several virulence genes did not change in the absence of SaeRS, amount of their corresponding proteins considerably decreased [47].

It has been proposed that *S. aureus* can be internalized within human epithelial cells. Garzoni and colleagues examined *S. aureus* transcription during internalization in to human epithelial cells. They showed that gene expression of extracellular and adherent *S. aureus* does not represent any changes when compared in the following conditions: cell culture medium, presence of cells, presence of cytochalasin D, and bacterial attachment to cells. On the other hand, gene expression showed remarkable changes after 2 and 6 hours of internalization [48]. In addition, *in vivo* *S. aureus* transcription studies have also indicated that *in vivo* virulence regulation differs from *in vitro* conditions [42]. In a recent study, changes of complete transcriptome of *S. aureus *during adaptation to the mouse lung environment were investigated [49]. In this study the evolution of transcription response of *S. aureus* during the first stages of pneumonia development, 6 hours after introduction to the airway, was compared with the stationary and early exponential phase in laboratory conditions. Results indicated that 50% of genes involved in amino acids biosynthesis were up-regualted *in vivo* (lung environment) compared to *in vitro* (laboratory) conditions, glycolysis genes were up-regulated *in vivo* while gluconeogenesis genes were down-regulated, genes involved in fermentation were generally down-regulated *in vivo*, almost all TCA cycle genes are down-regulated *in vivo*, 10 of 21 genes involved in electron transport system were down-regulated and almost 50% of the genes responsible for cofactor biosynthesis were down-regulated while 50% were up-regulated.

## HAEMOPHILUS INFLUENZAE

*Haemophilus influenzae *is a gram negative coccobacillus frequently found as upper respiratory tract flora in the human. It is notorious for causing otitis media in children. Recently it has been recognized that this bacteria has a dominant role in lower respiratory tract infections. However interactions between *H. influenzae *and host lung has not been completely elucidated. *H. influenzae* has the ability to growth under both aerobic and anaerobic conditions [50]. Heme is required for its growth under aerobic conditions [51]. *H. influenzae* has a small genome compared to other bacterial residents of lung (Table **1**). Based on the presence of polysaccharide capsule *H. influenzae* is divided in to two types: typeable (having capsule) and non-typeable *H. influenzae (*NTHi). Heterogeneity of NTHi group is very high and many subtypes have been recognized so far [52]. NTHi has also been recognized as the first lower airway bacterium in CF [53]. Despite this fact that *H. influenzae* genome was the first completely sequenced genome, it is somewhat surprising that not much work have been performed so far on its genome scale gene expression. In a study conducted in J. Craig Venter institute comparative genomics on twenty *H. influenzae* strains was carried out [54]. They developed a new microarray chip representing 4600 70mers Open Reading Frames (ORFs) including genes from strains KW20/RD, HK1212 (believed to cause Brazilian purpuric fever (BPF) or conjunctivitis) and new features belong to unfinished sequencing projects from NCBI. Their results indicated that with few exceptions, entire virulence genes previously described for *H. influenzae* are present in HK1212 strain. Moreover they showed that HK1212 genome has evolved to be more invasive in a capsule independent manner. In another study Gmuender and colleagues analyzed the transcriptional responses of *H. influenza* to DNA gyrase inhibitors; Novobiocin and Ciprofloxacin. Their results demonstrated that genes involved in DNA gyrase were up-regulated in the presence of both chemicals whereas topoisomerase I related genes were down-regulated. In addition it was indicated that ciprofloxacin can lead to SOS responses [55].

## CONCLUSION

Several pathogenic bacteria co-existing in human lung during severe lung diseases including COPD and CF are well-known examples of this kind of microbial societies. *P. aeruginosa*, *S. aureus*, *B. cenocepacia* and *H. influenzae* are the most notorious bacteria among lung pathogens that their corresponding infections gradually lead to deterioration of lung function and in some cases to death. In this study, we have reviewed the genome complexity and available data sources for four major pathogenic bacteria causing CF diseases (Table **1** and Table **2**). In addition, the orthologous genes of these bacteria have been compared and the number of common orthologous genes between understudied microorganisms were represented (Fig. **2**). A few studies addressing expressional responses of lung pathogens in isolation and under various conditions have been represented. We have focused on major studies where differentially expressed genes in CF lung conditions have been listed for each one of these bacteria. These studies could highlight the up-regulated and down-regulated genes (and biological processes) in each of the mentioned microorganisms during CF lung infection. Bacterial diseases in many cases are the outcome of complex interactions between a variety of pathogenic species, natural microflora and the host. As a consequence, gene expression in a pathogenic species is influenced by the presence of other pathogens as well as resident microflora within the host [7]. Some recent studies concerned with analyzing an individual pathogenic species gene expression in response to the presence of resident species in lung environment [56, 7]. However there is a lack of comprehensive studies portraying the pattern of gene expression in the lung pathogenic species in the presence of other pathogenic strains in the lung environment. Comparative transcriptomics studies such as detecting co-expression conservation can lead to identification of conserved co-expression modules (if exists) in these bacterial pathogens, and also can point up the differences in the variable part of the conserved modules (Fig. 3). As an example, several co-expression modules have been detected as highly conserved across Escherichia coli and Salmonella enterica [57], and some of these conserved co-expressed modules include some pathogenesis related genes which have been featured as S. enterica species-specific genes in the variable part of co-expressed modules. Similar studies can also be conducted over the bacterial strains causing CF lung infections to determine in which biological modules these bacteria employ their pathogenesis related genes. Perhaps the major difficulty to detect co-expression conservation across different bacterial strains involved in CF infections is the lack of sufficient expression data sources as a large collection of experimental conditions is needed to calculate relevant coexpression values across two genes. Such a data exists for well-studied model bacteria like E. coli and B. subtilis, but may not exists for most of the CF infections causing bacteria. Furthermore, by considering the fact that some interspecies cell-cell communications have been reported for CF bacteria, expressional investigation of genes involved in interspecies signaling will be valuable in deep understanding of synergistic or antagonistic relationships among microorganisms involved in co-infections. Besides, unraveling the shared biochemical processes in microorganisms present in microbial communities such as polymicrobial biofilms may be helpful in finding new potential drug targets.

## Figures and Tables

**Table 1.** Genome level information of the major bacteria isolated from lung infections.

Bacterial Species	Size of Genome (Mb)	Number of Genes	Number of Essential Genes	Number of Proteins
*P. aeruginosa* PAO1	6.26	5680	335	5569
*S. aureus* subsp. *aureus* N315	2.84	2694	653	2613
*B. cenocepacia J2315*	8.05588	7375	406*	7116
*H. influenzae* RDKW20	1.83	1765	642	1610

* Number of essential genes of *Burkholderia thailandensis* E264 which is in the Bcc family obtained from DEG database (http://tubic.tju.edu.cn/deg/).

**Table 2.** Available gene expression, protein and metabolic interaction networks data for four species.

Pathogen Species	Gene Expression Data in GEO	RNA-Seq	RNA-Seq (CF)	Protein-protein Interaction Data	Metabolic Network
*P. aeruginosa*	1339	372	74	[18]	[21]
*S. aureus*	2173	6191	4	[19]	[22]
*H. influenzae*	401	6	0	[20]	[23]
*B. cepacia complex*	81	81	1	-	[24]

**Table 3.** Comparison of three highly used gene expression technologies: Microarray, SAGE and RNA-Seq.

	Advantages	Disadvantages
Microarray SAGE RNA-Seq	- cost effective and space efficient - No need for prior knowledge of genes' sequences - Accuracy of mRNA quantitation in SAGE [32]. - No need for prior knowledge of genes' sequences - Highly efficient in detecting sequence variations such as SNPs in the transcribed regions as well as identification of precise transcription boundaries and isoforms [30]. - Covers the whole transcriptome	- Failure in recognizing low abundant transcripts - Presence of background hybridization and non specific hybridization - Unable to detect single nucleotide polymorphism or to measure the expression level of isoforms (alternatively spliced transcripts)[30]. - Prior knowledge of genes' sequences is essential [31]. - Unable to detect isoforms including alternatively spliced transcripts [30]. - High cost - Not usually performed for the whole transcriptome (just selected genes)
